# Research on the development efficiency of regional high-end talent in China: A complex network approach

**DOI:** 10.1371/journal.pone.0188816

**Published:** 2017-12-22

**Authors:** Zhen Zhang, Minggang Wang, Lixin Tian, Wenbin Zhang

**Affiliations:** 1 Nonlinear Scientific Research Center, Faculty of Science, Jiangsu University, Zhenjiang, Jiangsu, China; 2 School of Mathematical Science, Nanjing Normal University, Nanjing, Jiangsu, China; 3 Department of Mathematics, Nanjing Normal University Taizhou College, Taizhou, Jiangsu, China; 4 School of Mathematical Science, Taizhou Institute of Sci. & Tech., NUST, Taizhou, Jiangsu, China; Beihang University, CHINA

## Abstract

In this paper, based on the panel data of 31 provinces and cities in China from 1991 to 2016, the regional development efficiency matrix of high-end talent is obtained by DEA method, and the matrix is converted into a continuous change of complex networks through the construction of sliding window. Using a series of continuous changes in the complex network topology statistics, the characteristics of regional high-end talent development efficiency system are analyzed. And the results show that the average development efficiency of high-end talent in the western region is at a low level. After 2005, the national regional high-end talent development efficiency network has both short-range relevance and long-range relevance in the evolution process. The central region plays an important intermediary role in the national regional high-end talent development system. And the western region has high clustering characteristics. With the implementation of the high-end talent policies with regional characteristics by different provinces and cities, the relevance of high-end talent development efficiency in various provinces and cities presents a weakening trend, and the geographical characteristics of high-end talent are more and more obvious.

## Introduction

At present, high-end talent with scientific and technical knowledge, innovation and creativity has become the most valuable capital and core resources, it is the main source and factor of economic growth, and the level of high-end talent development is directly related to the power of a country or region. Science and technology creation is the source of economic growth, it can greatly improve the productivity, promote the transformation of economic growth mode and the continuous optimization of economic structure, and promote the national or regional economic development. High-end talent is an indispensable element in the promotion of regional innovation, and the size and quality of talent groups determine the operational efficiency of regional innovation system. Therefore, high-end talent has become one of the basic determinants of a region development, as well as the whole country development, while the cultivation and maintenance of regional science and technology talent innovation ability has become the key to the construction and improvement of regional innovation system. How to objectively and scientifically evaluate the ability of regional high-end talent innovation is of great significance to the formulation of a regional science and technology innovation strategy.

In recent years, scholars have carried out a large number of researches about the co-movement patterns between high-end talent and economic growth. Greiner A [1] and Hansen S et al [2] conducted a comparative study of the development of high-end talent between the United States and Germany. Abdih Y et al [3] empirically investigated the knowledge production function and intertemporal spillover effects using cointegration techniques. By second-generation endogenous growth models, Ang J B et al [4] explained the productivity trends and knowledge production in the Asian miracle economies. Fuller D [5] assessed the Role of High-End Talent in China by Denis Fred Simon and Cong Cao. By analyzing the common environment and individual environment required for high-end talent. Liu R et al [6] constructed the social and ecological environment evaluation system of high-end talent, including the environment of science and technology development, the environment of science and technology, the environment of open system, the harmonious environment and the living environment. Fang Y et al [7] analyzed the tripartite experiences among colleges and universities, enterprises and government in the United States, Germany and South Korea, and put forward some suggestions to high-end talent training in China. Based on the 2001–2010 data of high-end talent and the regional innovation ability. Rui X et al [8] studied the cointegration analysis, granger causality test and impulse analysis to the scale of high-end talent, effect of high-end talent and regional innovation ability respectively. Based on the scientific classification on high-end talent, the characteristics of different kinds of talents, including the fields of basic research and basic applied research, technology R&D and applications, innovation and entrepreneurship were analyzed by Zhao W et al [9]. Sheng N et al [10] constructed the evaluation system of high-end talent and put forward the construction process,index system and management process,based on the definition of high-end talent. In order to analyze the capability of regional high-end talent’s innovation. Shen C et al [11] established a multi-layer evaluating target system based on the various influential factors on capability of the regional technical innovation. Based on the Cork Douglas production function and the data from 1991 to 2010 of the 12 provinces and regions in China. Sun J et al[12] constructed multiple linear regression model and its revised model, and analyzed the influences of high-end talent on China's regional economic development. Li Z et al [13] studied the evaluation of the development efficiency of high-end talent in western China and its influencing factors. To get the development efficiency of high-end talent in 30 provinces and municipalities in China,Zhang C et al[14] used DEA method and established a Tobit model by setting these values of efficiency as the dependent variable and environmental factors as independent variables.

To sum up, the previous researches on high-end talent can be divided into two categories. One is the promotion of high-end talent development to economy, and the second is the evaluation of high-end talent development. Many scholars have made a lot of conclusions. In recent years, some scholars have gradually begun to pay attention to the evaluation of the development efficiency of regional high-end talent [13–14] in China, and they found some results. However, there are still several problems in the existing research: (1) The existing research is just to select the data for a certain period of time, and the evaluation of regional high-end talent development efficiency is static. In fact, the efficiency of regional high-end talent development changes with time. Whether this change has a certain regularity, it is failed to cover this aspect in previous studies. (2) The existing researches only obtained the evaluation of the development efficiency about high-end talent in different regions at a certain time, but lacked the correlation analysis among these results. The association evolves over time, the role of different regions in the high-end talent development system can be obtained by analyzing the evolution of the association. In order to solve the above problems, this paper will use complex network theory [15–24] to study the high-end talent development. First of all, the annual assessment of the regional high-end talent development efficiency in China from 1990 to 2015 is constructed, and the evaluation matrix of regional high-end talent development efficiency is established. Then, with the help of complex network theory, the regional development efficiency evaluation matrixes are converted into a series of complex networks. Finally, the dynamic topology indicators of the networks are defined, and the spatial evolution characteristics of the regional high-end talent development efficiency are analyzed.

## Methodology

### The regional high-end talent development efficiency matrix

The efficiency of the high-end talent development is an important index to evaluate the development level of science and technology talents, select the appropriate input and output indicators, using the scientific method has important practical significance for the efficiency evaluation of the regional high-end talent development. The efficiency of regional high-end talent development can be calculated by DEA method, and the calculation process is as follows: Assume the high-end talent evaluation system has a total of *n* evaluation objects (i.e. decision-making unit *DMU*), and the evaluation index system consists of *m* input indicators and *s* output indicators, where the input and output vectors of the *j*th in *DMU* are

$$X_{j}={\left(x_{1j},x_{2j},\cdots ,x_{mj}\right)}^{T}>0,\;Y_{j}={\left(y_{1j},y_{2j},\cdots ,y_{sj}\right)}^{T}>0,\;j=1,2,\cdots ,n$$

The *C*^2^*R* model with non-Archimedes infinity *ε* for evaluating for the *k*th decision-making unit is:

$$\left\{ \begin{array}{l} \max\;\mu^{T}Y_{k}\\ s.t.\\ \omega^{T}X_{j}-\mu^{T}Y_{j}\geq 0,j=1,2,\cdots ,n\\ \omega^{T}X_{k}=1\\ \omega \geq \varepsilon \hat{e},\mu \geq \varepsilon e,\hat{e}={\left(1,1,\cdots ,1\right)}^{T}\in E_{m},e={\left(1,1,\cdots ,1\right)}^{T}\in E_{s} \end{array}\right.$$ (1)

The dual plan is as follows:

$$\left\{ \begin{array}{l} \min\;[h-\varepsilon \left({\hat{e}}^{T}s^{-}+e^{T}s^{+}\right)]\\ s.t.\\ \sum_{j=1}^{n}\lambda_{j}X_{j}+s^{-}=hX_{k}\\ \sum_{j=1}^{n}\lambda_{j}Y_{j}-s^{+}=Y_{k}\\ \lambda_{j}\geq 0,j=1,2,\cdots ,n,s^{-}\geq 0,s^{+}\geq 0 \end{array}\right.$$ (2)

where *s*^+^ and *s*^−^ are the relaxation variables and residual variables respectively,and the optimal solution *h*_*k*_ represents the *k*th efficiency value of *DMU* in (2).

The *k*th efficiency value *h*_*tk*_ of *DMU h*_*tk*_ can be calculated at different times *t*, thus the high-end talent development efficiency matrix *H* = [*h*_*tk*_]_*T*×*n*_ will be obtained.

### Window division

Based on the analysis of the instruction, the development efficiency of regional high-end talent is not a constant, but varies with time. Therefore, only evaluating the development efficiency of regional high-end talent at different time can the comprehensive development of regional high-end talent be fully reflected. In order to achieve this motivation, we need to divide the whole sample time into continuous small time segments, thus we introduce the method of window division. The idea of window division is to divide the data matrix into several blocks, and these blocks are interconnected to transform the static data matrix into dynamic analysis. The window division process consists of two steps:

#### Step1: Determine the window length *L*

Considering the *M* × *N*-dimensional data, and let *L* be the selected window length, then a new *L* × *N*-dimensional data matrix $R_{L\times N}^{\left(t\right)}$ can be formed in each window according to the random matrix theory, where *M*, *N* are large numbers, and *M* > *L* > *N*.

#### Step2: Determine the sliding step *l*

**Fig 1:**
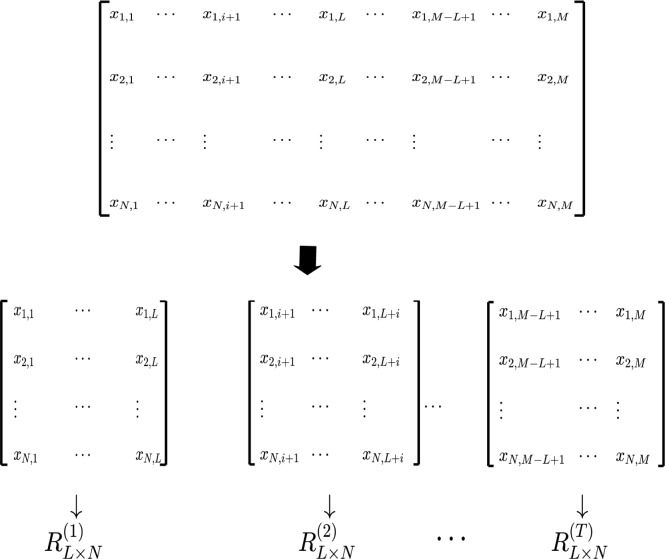
The window division process.

In order to ensure the connection between the windows, the sliding step *l* needs to meet the condition *l* ≤ *L*. After the sliding step *l* is selected, $T=\left[\frac{M-L}{l}+1\right]$ windows and a row of *L* × *N*-dimensional data matrix $R_{L\times N}^{\left(t\right)}$ can be obtained, where *t* = 1,2,⋯,*T*. Then, the complex networks are built for each data matrix and *T* networks *NC*^(*t*)^ are obtained, where *t* = 1,2,⋯,*T*. The window division process is shown in Fig 1.

### Construction and characterization of global dynamics

After the sliding window construction is completed, we need to transform the data matrix $R_{L\times N}^{\left(t\right)}$ into complex network *NC*^(*t*)^ in each sliding window. The process of constructing complex network and extracting the global dynamic characteristics from the network mainly includes the following three steps:

#### Step1: Calculate the correlation coefficient matrix in each time window *t*

According to the data matrix $R_{L\times N}^{\left(t\right)}$ in the time window *t*, the correlation matrix $C_{N\times N}^{{}^{\left(t\right)}}$ can be calculated:

$$C_{N\times N}^{{}^{\left(t\right)}}=\left(\begin{array}{cccc} C_{11}^{\left(t\right)} & C_{12}^{\left(t\right)} & \cdots & C_{1N}^{\left(t\right)}\\ C_{21}^{\left(t\right)} & C_{22}^{\left(t\right)} & \cdots & C_{2N}^{\left(t\right)}\\ \vdots & \vdots & \vdots & \vdots \\ C_{N1}^{\left(t\right)} & C_{N1}^{\left(t\right)} & \cdots & C_{N,N}^{\left(t\right)} \end{array}\right)$$ (3)

where $C_{ij}^{\left(t\right)}$ is an element of the matrix $C_{N\times N}^{{}^{\left(t\right)}}$, *i* = 1,2,⋯,*N*, *j* = 1,2,⋯,*N*. $C_{ij}^{\left(t\right)}$ means the correlation coefficient between the vectors $X_{i}^{\left(t\right)}$ and $X_{j}^{\left(t\right)}$, and the calculation formula is as follows:

$$C_{ij}^{\left(t\right)}=\frac{\sum_{k=1}^{L}\left[X_{i}^{\left(t\right)}\left(k\right)-\left\langle X_{i}^{\left(t\right)}\right\rangle \right]\cdot \left[X_{j}^{\left(t\right)}\left(k\right)-\left\langle X_{j}^{\left(t\right)}\right\rangle \right]}{\sqrt{\sum_{k=1}^{L}{\left[X_{i}^{\left(t\right)}\left(k\right)-\left\langle X_{i}^{\left(t\right)}\right\rangle \right]}^{2}}\cdot \sqrt{\sum_{k=1}^{L}{\left[X_{j}^{\left(t\right)}\left(k\right)-\left\langle X_{j}^{\left(t\right)}\right\rangle \right]}^{2}}}$$ (4)

where $X_{i}^{\left(t\right)}\left(k\right)$ and $X_{j}^{\left(t\right)}\left(k\right)$ are the elements of the vector $X_{i}^{\left(t\right)}$ and $X_{j}^{\left(t\right)}$ respectively, and $\left\langle X_{i}^{\left(t\right)}\right\rangle =\sum_{k=1}^{L}X_{i}^{\left(t\right)}\left(k\right)/L$, $\left\langle X_{j}^{\left(t\right)}\right\rangle =\sum_{k=1}^{L}X_{j}^{\left(t\right)}\left(k\right)/L$, *k* = 1,2,⋯,*L*.

**Remark:** In some cases, it is also possible to determine the relationship between the vectors $X_{i}^{\left(t\right)}$ and $X_{j}^{\left(t\right)}$ according to the distance between vectors, and the formula between vectors $X_{i}^{\left(t\right)}$ and $X_{j}^{\left(t\right)}$ is as follows:

$$d_{ij}^{\left(t\right)}=\sum_{k=1}^{L}\left\|X_{i}^{\left(t\right)}\left(k\right)-X_{j}^{\left(t\right)}\left(k\right)\right\|$$

#### Step2: Build the adjacency matrix in each time window *t*

To establish the complex network, the key is to determine the corresponding adjacency matrix. In each window, the correlation coefficient matrix ***C***^(*t*)^ can be converted into adjacency matrix ***A***^(*t*)^ of the corresponding complex network *NC*^(*t*)^ as follows:

$$A_{ij}^{\left(t\right)}=\Theta \left(\left|C_{ij}^{\left(t\right)}\right|-r_{c}\right),\;\Theta \left(x\right)=\{\begin{array}{cc} 1 & ,x\geq 0\\ 0 & ,x<0 \end{array}$$ (5)

where $A_{ij}^{\left(t\right)}\in A^{\left(t\right)}$, Θ(*x*) is Heaviside function and *r*_*c*_ is the threshold. Owing to the edge number of the network decreases as the threshold *r*_*c*_ increases, the selection of thresholds *r*_*c*_ is important. If the value of *r*_*c*_ is too small, then the relatively weak nodes may be connected, and the degree of distinction will be greatly reduced. If the value of *r*_*c*_ is too large, it may miss some relevant nodes, the obtained network structure will be too simple. So choosing an appropriate threshold *r*_*c*_ is critical to build the network. The selected method of the thresholds *r*_*c*_ in this paper is as follows:

$$r_{c}=\sum_{t=1}^{T}\left\langle C^{\left(t\right)}\right\rangle /T,\;\left\langle C^{\left(t\right)}\right\rangle =\sum_{j=1}^{N}\sum_{i=1}^{N}\left|C_{ij}^{\left(t\right)}\text{-}I_{ij}\right|/\left(N\times N\right)$$ (6)

where *I*_*ij*_ ∈ ***I***, and ***I*** represents a *N*-order unit matrix. To build a complex network in practical applications, let the threshold *r*_*c*_ float up and down according to the actual needs is a good way.

#### Step3: Extract the global dynamics of network sequences

According to the complex networks *NC*^(*t*)^ which are obtained in step2, the distance between the networks will be calculated by the adjacency matrixes of the networks to describe the global dynamic characteristics of the system, and the formula is as follows:

$$d\left(A^{\left(t_{1}\right)},A^{\left(t_{2}\right)}\right)=\sum_{i>j}\left\|A_{ij}^{\left(t_{1}\right)}-A_{ij}^{\left(t_{2}\right)}\right\|$$ (7)

Combining with the appropriate threshold *r*_*a*_, the evolution diagram between the networks can be obtained by the following binary matrix:

$$R_{t_{1},t_{2}}\left(A^{\left(t\right)}\right)=\{\begin{array}{cc} 1, & d\left(A^{\left(t_{1}\right)},A^{\left(t_{2}\right)}\right)<r_{a}\\ 0, & otherwise \end{array}$$ (8)

In this way, we can draw the 2-dimentional relationship figures (abscissa and ordinate are time t) between networks, and describe the global dynamic characteristics of high-dimensional time series system by the relationship figures.

### Dynamic indicators

Based on the method of section 2.3, the *M* × *N*-dimensional time series data can be transformed into $T=\left[\frac{M-L}{l}+1\right]$ complex networks *NC*^(*t*)^, *t* = 1,2,⋯,*T*. Each network has its own characteristic structure, and the feature structure changes with the time window *t*. Therefore, according to the topology structure of complex network, the network characteristics that change over time windows can be obtained. As these networks are undirected networks, the following characteristics are analyzed:

**The degree of network node at time** *t*, which is marked as $k_{i}^{\left(t\right)}$, it indicates the number of adjacent edges of the node $v_{i}^{\left(t\right)}$ at time *t*. Then the average degree of the network at time *t* is:

$$\left\langle k^{\left(t\right)}\right\rangle =\frac{1}{N}\sum_{i=1}^{N}k_{i}^{\left(t\right)}$$ (9)

**The clustering coefficient of network node at time** *t*, noted as $l_{i}^{\left(t\right)}$:

$$l_{i}^{\left(t\right)}=\frac{2E_{i}^{\left(t\right)}}{k_{i}^{\left(t\right)}\left(k_{i}^{\left(t\right)}-1\right)}$$ (10)

then the average clustering coefficient of network at time *t* is $\left\langle l^{\left(t\right)}\right\rangle =\frac{1}{N}\sum_{i=1}^{N}\frac{2E_{i}^{\left(t\right)}}{k_{i}^{\left(t\right)}\left(k_{i}^{\left(t\right)}-1\right)}$, where $E_{i}^{\left(t\right)}$ is the number of edges that actually exist between $k_{i}^{\left(t\right)}$ nodes which are directly connected to the node $v_{i}^{\left(t\right)}$ at time *t*.

**The betweenness of network node at time** *t*, **noted as** $B_{i}^{\left(t\right)}$,

$$B_{i}^{\left(t\right)}=\sum_{j\neq l\neq i}\frac{g_{jl}^{\left(t\right)}\left(i\right)}{g_{jl}^{\left(t\right)}}$$ (11)

then the average betweenness of networks at time *t* is $\left\langle B^{\left(t\right)}\right\rangle =\frac{1}{N}\sum_{i=1}^{N}\sum_{j\neq l\neq i}\frac{g_{jl}^{\left(t\right)}\left(i\right)}{g_{jl}^{\left(t\right)}}$, where $g_{jl}^{\left(t\right)}$ represents the number of the shortest paths between nodes $v_{j}^{\left(t\right)}$ and $v_{l}^{\left(t\right)}$ at time *t*, and $g_{jl}^{\left(t\right)}\left(i\right)$ represents the number of the shortest paths between nodes $v_{j}^{\left(t\right)}$ and $v_{l}^{\left(t\right)}$ which pass the node $v_{i}^{\left(t\right)}$.

The average distance of network at time *t*, noted as ⟨*L*^(*t*)^⟩:

$$\left\langle L^{\left(t\right)}\right\rangle =\frac{2}{N\left(N-1\right)}\sum_{i=1}^{N}\sum_{j=i+1}^{N}d_{ij}^{\left(t\right)},$$ (12)

where $d_{ij}^{\left(t\right)}$ represents the distance between nodes $v_{j}^{\left(t\right)}$ and $v_{l}^{\left(t\right)}$ at time *t*.

The network topology indicators involved in the complex network can be extended to the dynamic indicators, the needs of the research objects can be selected, and the indicators are not listed here.

## Empirical analysis

### Data sources

Based on the statistical data of 31 provinces from January 1990 to December 2015 in China, this paper analyze the factors of development efficiency of scientific and technological talent evaluation and efficiency, and all data from the China statistical yearbook on science and technology (1990–2016). All data files are available from the National Bureau of Statistics of the People’s Republic of China database.(http://www.stats.gov.cn/ztjc/ztsj/kjndsj/#). The selected provinces and cities as show in Table 1.

**Table 1:** The selected provinces and abbreviation.

NO.	Province	Abbreviation	NO.	Province	Abbreviation	NO.	Province	Abbreviation
1	BeiJing	BJ	12	AnHui	AH	23	SiChuan	SC
2	TianJin	TJ	13	FuJian	FJ	24	GuiZhou	GZ
3	HeiBei	HE	14	JiangXi	JX	25	YunNan	YN
4	ShanXi	SX	15	ShanDong	SD	26	XiZang	XZ
5	NeiMengGu	NM	16	HeNan	HA	27	ShanXi	SN
6	LiaoNing	LN	17	HuBei	HB	28	GanSu	GS
7	JiLin	JL	18	HuNan	HN	29	QingHai	QH
8	HeiLongJiang	HL	19	GuangDong	GD	30	NingXia	NX
9	ShangHai	SH	20	GuangXi	GX	31	XinJiang	XJ
10	JiangSu	JS	21	HaiNan	HI			
11	ZheJiang	ZJ	22	ChongQing	CQ			

### Evaluation of regional high-end talent development efficiency

**Fig 2:**
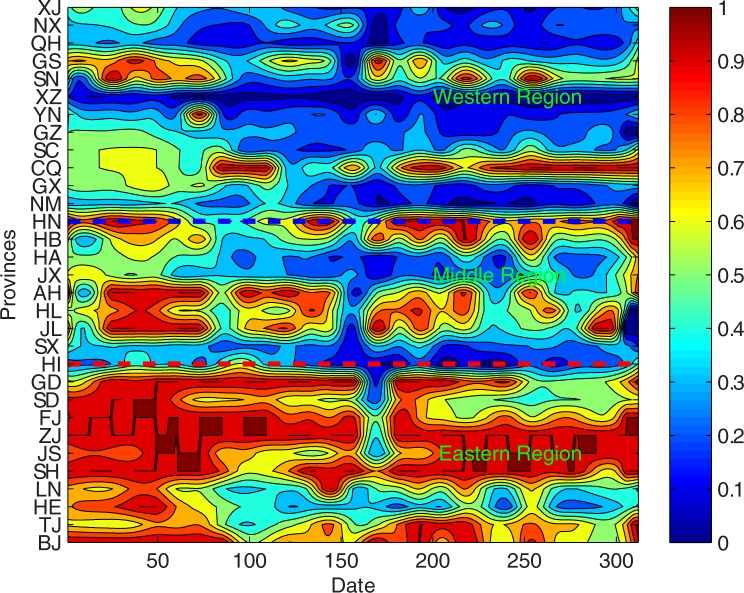
The evolution image of regional high-end talent development efficiency.

During the process of DEA efficiency evaluation, four indicators from each province: research and development institutions R & D number, R & D personnel, research funding, research and development institutions R & D project input number are selected as input indicators; In addition, three indicators: the number of patent applications, patents Authorization number and the number of Chinese science and technology papers included in foreign database are selected as output indicators. Based on the DEA model, the evolution image of regional high-end talent development efficiency is obtained from 1990 to 2015, as shown in Fig 2.

According to the empirical results of the high-end talent development efficiency of provinces and cities (See Fig 2), the average efficiency value of the top five provinces and cities are Zhejiang, Shanghai, Guangdong, Fujian and Jiangsu, their corresponding average efficiency are 0.9734, 0.9898, 0.8457, 0.844 and 0.8322, respectively. And the last five are Xinjiang, Yunnan, Inner Mongolia, Qinghai and Tibet, their corresponding average efficiency are 0.2847, 0.2806, 0.2252, 0.1813 and 0.0612, respectively. From the regional location, the average efficiency value of the top provinces and cities are from the eastern region, while the provinces and cities with the lower rankings are from the western region. According to the calculation results in Fig 2, the average efficiency value of high-end talent development in the eastern region is 0.7226, the average efficiency of high-end talent development in the central and western regions are respectively 0.5580 and 0.3558. Therefore, the average efficiency of high-end talent development in the western region is at a low level among the three regions, especially in Sichuan, Guangxi, Guizhou, Ningxia, Xinjiang, Yunnan, Inner Mongolia, Qinghai and Tibet, and their average score for high-end talent development is less than 0.5.

### Network construction and global feature analysis

Based on the selected data and the method of sliding window construction, we establish the corresponding sliding window. Combining *N* = 31 with the method of sliding window construction in section 2.2, we set *L* = 120 and *l* = 1, then get $T=\left[\frac{M-L}{l}+1\right]=\left[\frac{312-120}{1}+1\right]=193$ sliding windows with ten years for a period of time. To determine the threshold *r*_*c*_, we calculate the correlation coefficient matrix among the 31 provinces of high-end talent development efficiency in each sliding window by the formulas (3) and (4), and get the threshold *r*_*c*_ = 0.3849 by (2.6).

**Fig 3:**
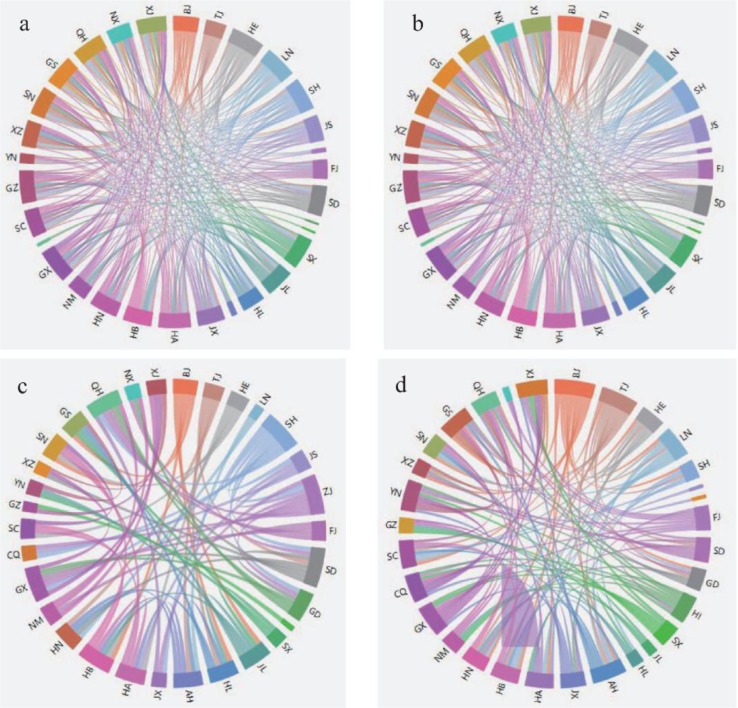
The network structure of national high-end talent development efficiency at different time window. (a) December 2000. (b) December 2006. (c) December 2010. (d) December 2015.

According to the network construction method, the network structure of national high-end talent development efficiency in each window at time *t*, as well as 193 networks can be obtained. The network structure images of the December 2000, December 2006, December 2010 and December 2015 are as shown in Fig 3A, 3B, 3C and 3D.

**Fig 4:**
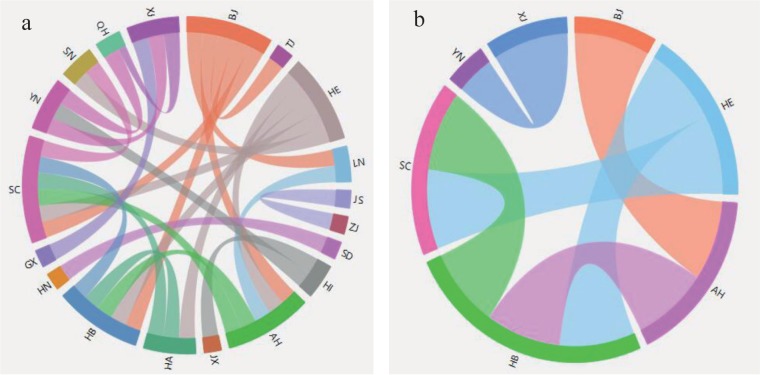
The effect of threshold changes on network structure. (a) *r*_*c*_ = 0.6849. (b) *r*_*c*_ = 0.8849.

It can be seen from Fig 3 that there are different structures for the network in different time windows, and the dynamic characteristics of the research object can be revealed by the changes of the network structure. In Section 2.3, we have already explained that the choice of thresholds *r*_*c*_ has a significant effect on the network structure. In the case of the network structure in December 2015 (Fig 3D), let *r*_*c*_ = 0.6849 and *r*_*c*_ = 0.8849, respectively, then the new network structures are obtained (See Fig 4A and 4B).

Comparing the network structure in Fig 4A and 4B and Fig 3D, it can be found that the network edges in Fig 3D and Fig 4A are 117 and 40 respectively, and there are only 6 in Fig 4D. Therefore, the smaller the threshold *r*_*c*_, the more connections in the network, and the greater *r*_*c*_, the less the connection in the network. Therefore, choosing the appropriate threshold is the key to the rational analysis of the system.

Next, we will analyze the global dynamic characteristics of the regional high-end talent development efficiency network. For the sake of observation, we compare the regional high-end talent development efficiency network with the random network generated by random time series.

**Fig 5:**
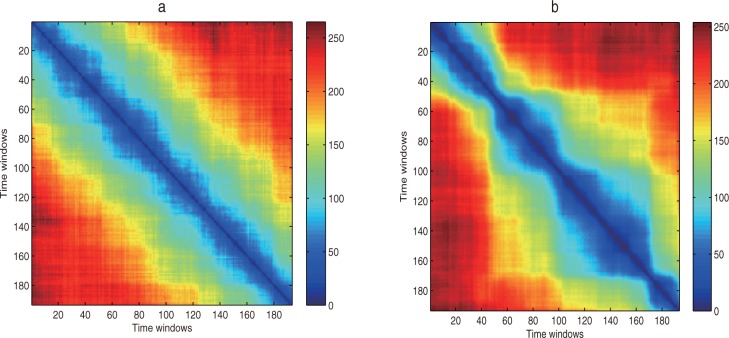
The global dynamic characteristics. (a) Random network. (b) Regional high-end talent development efficiency network.

According to the method of the complex network in section 2,the network generated by the random time series can effectively reflect the global dynamical properties of the random time series. We compare the topological structure of the regional high-end talent development efficiency network with the random network, and find out the differences of their topological structures, thus we can reveal the unusual global dynamic characteristics of the regional high-end talent development efficiency network. We take the same threshold *r*_*a*_ = 10 to calculate the distance between these two networks under different time windows by (7), and construct the binary matrix by (8), then the evolution diagrams are obtained (See Fig 5A and 5B).

Comparing the evolution diagram of the random network with the that of regional high-end talent development efficiency network in Fig 5A and 5B, it can be seen that the blue area is located near the diagonal in Fig 5A, indicating the random networks with the high similarity appear relatively close to the time, while the random networks with a smaller similarity appear a long time apart (The blue area does not appear in addition to the vicinity near the diagonal in Fig 5A). Thus the random networks generated by random time series only have a short-range correlation in the evolution process and do not have a long-range correlation. For the network of the national regional high-end talent development efficiency, the blue area is mainly distributed in the vicinity of the diagonal before 2005 in Fig 5B, indicating that during the evolution process before 2005, the regional high-end talent development efficiency network only has the short-range correlation. However, the blue area of the distribution becomes complicated after 2005 in Fig 5B, it spreads from the diagonal area, revealing that the national high-end talent development efficiency network has both a short-range correlation and a long-range correlation during the evolution process after 2005.

### Dynamic characteristics

Many concepts and methods have been proposed to describe the statistical characteristics of complex network structures, such as degree distribution, characteristic path length, clustering coefficient, betweenness, joint degree distribution, assortativity coefficient, coreness, closeness centrality, centrality, eigenvector centrality, etc. Owing to degree distribution, characteristic path length, clustering coefficient and betweenness are four basic concepts to describe the statistical characteristics of complex networks, these four indexes are choosen to describe the evolution characteristics of the regional high-end talent development efficiency.

#### The evolution of node degree

**Fig 6:**
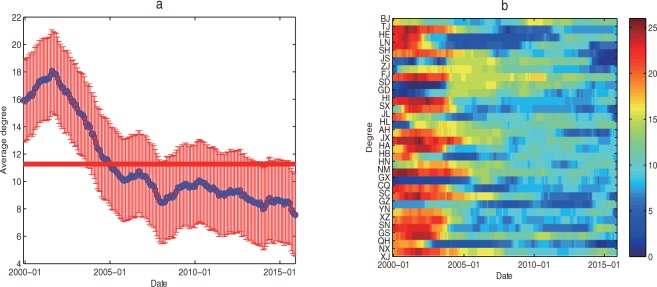
The evolutionary image of the degree. (a) The national average degree at time *t*. (b) The degree of each province and city at time *t*.

The evolutionary image of the average degree at time *t* about each province and city is calculated by formula (9) (See Fig 6A) based on the national regional high-end talent development efficiency network. What’s more, the evolution image of the degree about each province and city is shown in Fig 6B.

According to the complex network theory, the size of the node degree reflects the number of neighbors of the node [22]. In the development efficiency evaluation of the national regional high-end talent, the degree of province and city can be interpreted as the level of synchronization. The greater the degrees of the provinces and cities, the country's higher average, indicating that the higher synchronization level of the national regional high-end talent development efficiency. It can see from Fig 6A that, the national average degree showed a trend of first rise and then decline in the macro level during the period from December 1999 to December 2015. That is to say, the synchronization level of regional high-end talent development efficiency increased firstly and then descended. Among them, the average degree showed an upward trend from 15.9355 to 18.0645 in December 1999—August 2001, while it began to decline since August 2001. The average degree in January 2005 began to fall below the previous year's average of 11.2686 (shown by the red solid line in Fig 6A), then declined slowly, and the average degree reduced to 7.5484 in December 2015. Therefore, the national high-end talent development efficiency in the level of synchronization was showing a weakening trend, with the policies were introduced by provinces and cities in favor of high-end talent. From a micro perspective, it can be seen from Fig 6B that the evolution the degrees of provinces and cities are showing roughly the same downward trend, but they are different in details, which are mainly reflected in the following two aspects: (1) The differences of the degrees among provinces and cities are significant in the development efficiency network of the national regional high-end talent at the same time. For example, in December 1999 (See Fig 3A), there are the six degrees are more than 24: Hebei, Liaoning, Shanghai, Henan, Guangxi and Guizhou, and there are two degrees are only 1: Guangdong and Hainan. (2) In the evolution process of development efficiency network of the national regional high-end talent, the difference of the degree among the same province is remarkable. For example, the degree of Liaoning Province is 24 in December 1999 (See Fig 3A), while it is 2 in December 2010. On the whole, the rank of average degree about the national provinces and cities over the years are shown in Table 2.

**Table 2:** The rank of average degree about the national provinces and cities over the years.

provinces	BJ	TJ	HE	LN	SH	JS	ZJ	FJ
<*k*>	12.44041	11.66839	11.51813	8.704663	9.336788	11.83938	9.974093	11.54404
rank	7	13	16	28	26	12	23	15
provinces	SD	GD	HI	SX	JL	HL	AH	JX
<*k*>	16.03109	9.450777	9.326425	12.32124	10.31606	10.3886	11.49741	12.31088
rank	1	25	27	8	22	21	17	9
provinces	HA	HB	HN	NM	GX	CQ	SC	GZ
<*k*>	14.54922	12.09845	9.725389	12.15026	14.89637	6.678756	10.68912	13.25907
rank	3	11	24	10	2	31	20	6
provinces	YN	XZ	SN	GS	QH	NX	XJ	
<*k*>	7.103627	11.57513	11.3886	11.11399	14.01554	7.119171	14.29534	
rank	30	14	18	19	5	29	4	

It can be seen from Table 2 that the range of average degrees among provinces and cities over the years in the country is large, namely 6.6788–16.0311, which indicates that there exists obvious regional characteristics in the national high-end talent development efficiency. The top five provinces and cities according to the change of the average degree are Shandong, Guangxi, Henan, Xinjiang and Qinghai. The last five provinces and municipalities according to the average degree are Hainan, Liaoning, Ningxia, Yunnan and Chongqing. From the perspective of the three regions of country, the average degree of the central region is the largest (11.6509), the West is the second (11.1904), and the eastern region is the smallest (11.0758).

#### Evolutionary characteristics of clustering coefficient

**Fig 7:**
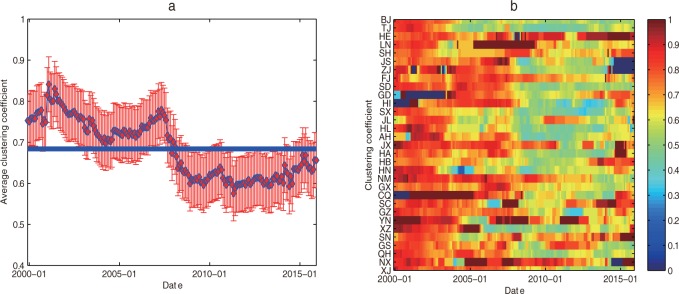
The evolutionary image of the clustering coefficient. (a) The national average clustering coefficient at time *t*. (b) The clustering coefficient of each province and city at time *t*.

The evolutionary image of the average clustering coefficient of provinces and cities is calculated (See Fig 7A) using the formula (10), and the evolutionary image of the clustering coefficient is shown in Fig 7B.

According to the complex network theory, the clustering coefficient refers to the probability that the two nodes which connected to the same node in the network are also connected to each other. This clustering coefficient is usually used to characterize the local structural features of the network [22]. In fact, the clustering coefficient reflects the tightness of a group (complete map) composed of provinces and cities. The country's average clustering coefficient reflects the overall trend of a whole nation's provinces and cities, that is, the trend of synchronization. Firstly, from a macro perspective, the average clustering coefficient in each time window is 0.6839 (Shown by the blue solid line in Fig 7A). That is to say, the synchronization trend of the regional high-end talent development efficiency is high. At the same time, it can be seen that the evolution trend of the national average clustering coefficient (See Fig 7A) has a similar shape to that of the national average degree (See Fig 6A). Then the provinces with larger degree often have a larger concentration factor in the national regional high-end talent development efficiency network. From a microscopic perspective, there are obvious differences between the evolutionary characteristics of the clustering coefficients and the degree (See Fig 6B) of different provinces (See Fig 7B) with times in some respects. In addition, it is mainly reflectd in two aspects: (1) Part of the provinces with small degrees show large concentration factors. For example, according to the national regional high-end talent development efficiency network structure (See Fig 3D), the national average degree reached to a minimum 7.5484 in December 2015. However, the clustering coefficients are 1 in the local area (Shown in the red symbol), such as Jilin (Degree is 3), Shaanxi (Degree is 7), Ningxia (Degree is 2), which means there exists a good synchronization. (2) Part of the provinces with high degrees show small concentration factors. For instance, the top three provinces according to the degree are Beijing, Tianjin and Anhui, their clustering coefficients are 0.5513, 0.5455 and 0.6545 respectively, and the clustering coefficients are less than the average of the calendar year 0.6839(See Fig 3D). They did not form a better synchronization area although their own degrees are large. Therefore, the regional characteristics of national high-end talent development efficiency can be analyzed by the evolution characteristics of the clustering coefficient of provinces and cities across the country. On the whole, the rank of clustering coefficient about the provinces and cities over the years during the evolution process of the national regional high-end talent development efficiency network are shown in Table 3.

**Table 3:** The rank of clustering coefficient about the provinces and cities over the years.

provinces	BJ	TJ	HE	LN	SH	JS	ZJ	FJ
<l>	0.612141	0.578611	0.819552	0.764366	0.707761	0.622711	0.614551	0.733854
rank	27	31	1	5	11	23	26	9
provinces	SD	GD	HI	SX	JL	HL	AH	JX
<l>	0.662486	0.589084	0.617795	0.583234	0.659688	0.609821	0.641138	0.770027
rank	20	29	25	30	21	28	22	4
provinces	HA	HB	HN	NM	GX	CQ	SC	GZ
<l>	0.663616	0.67216	0.618822	0.759443	0.69206	0.710297	0.75431	0.69364
rank	19	16	24	6	14	10	7	13
provinces	YN	XZ	SN	GS	QH	NX	XJ	
<l>	0.808598	0.664792	0.73718	0.700042	0.667026	0.787473	0.685784	
rank	2	18	8	12	17	3	15	

As can be seen from Table 3, the range of clustering coefficient among provinces and cities over the years in China varies from 0.5786 to 0.8196, and the average clustering coefficient is relatively high, indicating that the development of regional high-end talent has high gathering characteristics. The top five provinces and cities in view of average clustering are Hebei, Yunnan, Ningxia, Jiangxi and Liaoning. And the last five provinces are Beijing, Heilongjiang, Guangdong, Shanxi and Tianjin. From the three regions of country, the average clustering coefficient of the western region is the largest (0.7217), the east is the second (0.6657), and the central region is the smallest (0.6523).

#### Characteristics of the betweenness and characteristic path length

**Fig 8:**
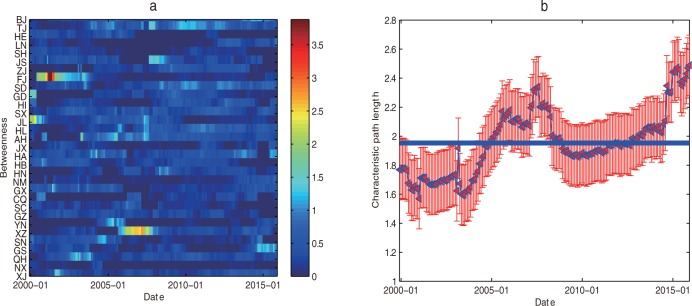
The evolutionary image of the betweenness and the characteristic path length. (a) The betweenness evolution image of each province. (b) The evolution image of the characteristic path length.

Using the formula (11), the betweenness evolution image of provinces and cities is calculated (See Fig 8A). According to the complex network theory, the betweenness is a global feature, reflecting the influence of the node or edge in the whole network. Based on the evolution process of the national regional high-end talent development efficiency network, we can identify the important provinces and cities in the national regional high-end talent development efficiency network with time. And the provinces and cities which play an important intermediary role national in regional high-end talent development efficiency network at any time can be found from Fig 8A. Besides, the rank of average betweenness of provinces and cities over the years is shown in Table 4.

**Table 4:** The rank of average betweenness of the provinces and cities over the years.

provinces	BJ	TJ	HE	LN	SH	JS	ZJ	FJ
<*B*>	0.455448	0.600902	0.18258	0.169543	0.152592	0.36458	0.274802	0.468415
rank	7	1	25	26	30	12	20	6
provinces	SD	GD	HI	SX	JL	HL	AH	JX
<*B*>	0.509667	0.260283	0.299541	0.437858	0.38709	0.448356	0.528409	0.161087
rank	4	22	16	9	11	8	2	28
provinces	HA	HB	HN	NM	GX	CQ	SC	GZ
<*B*>	0.489525	0.309939	0.261931	0.166861	0.349741	0.1888	0.188424	0.275452
rank	5	15	21	27	14	23	24	19
provinces	YN	XZ	SN	GS	QH	NX	XJ	
<*B*>	0.159118	0.524635	0.279772	0.280196	0.413724	0.052612	0.358118	
rank	29	3	18	17	10	31	13	

It can be seen from Table 3 that the range of average betweenness of provinces and cities is 0.0526–0.6009, and there are 14 provinces and cities more than the average number (0.3226). They are separately Tianjin, Anhui, Tibet, Shandong, Henan, Fujian, Beijing, Heilongjiang, Shanxi, Qinghai and Jilin according to the betweenness from high to low. Among them, five of these provinces and cities come from the eastern region, five from the central region and four from the western region. These provinces and cities play an important intermediary role in the regional high-end talent development efficiency network. From the view of three regions of the country, the average betweenness in the central region is the largest (0.3780), the second one is the East (0.3399), the western region is the smallest (0.2698).

The evolutionary image of the characteristic path length between the provinces and the provinces is calculated by the formula (12) (See Fig 8B), and it can be seen from Fig 8B that the average path length of the network increases gradually over time. Thus, the distance between the nodes in the network shows that, the geographical characteristics of high-end talent efficiency are more and more obvious with the regional characteristics of high-end talent policies were introduced by provinces and cities.

## Conclusion

In this paper, according to the panel data of 31 provinces and cities from 1991 to 2016 in China, the regional development efficiency matrix of high-end talent is obtained by DEA method, and the matrix is converted into a continuous change of complex networks through the construction of sliding window, then the characteristics of regional high-end talent development efficiency system are analyzed with a series of continuous changes in the complex network topology statistics. 193 networks of national regional high-end talent development efficiency are constructed, and the dynamic indexes of the networks are calculated such as the degree of nodes, the clustering coefficient, the betweenness, the average path length and so on. And we obtain the following conclusions:

1. The average efficiency of high-end talent development in the eastern region is 0.7226, and the average efficiency in the central region and the western region are 0.5580 and 0.3558, respectively. Therefore, the average efficiency of high-end talent development in the western region is at a low level, especially in Sichuan, Guangxi, Guizhou, Ningxia, Xinjiang, Yunnan, Inner Mongolia, Qinghai and Tibet, they are less than 0.5.
2. The range of average degrees of provinces and cities over the years in the country is 6.6788–16.0311, and the range is large, which indicates that the national high-end talent development efficiency has obvious regional characteristics. The top five provinces and cities in terms of the change of the average degree are Shandong, Guangxi, Henan, Xinjiang and Qinghai. And the last five provinces and cities are Hainan, Liaoning, Ningxia, Yunnan and Chongqing. From the three regions of country, the average degree of the central region is the largest (11.6509), the West is the second (11.1904), and the eastern region is the smallest (11.0758).
3. Over the years, the clustering coefficient of provinces and cities in China varies from 0.5786 to 0.8196, and the average clustering coefficient is relatively high, indicating that the regional high-end talent development has high gathering characteristics. The top five provinces and municipalities given the average clustering coefficient are Hebei, Yunnan, Ningxia, Jiangxi and Liaoning. And the last five provinces are Beijing, Heilongjiang, Guangdong, Shanxi and Tianjin. From the three regions of country, the average concentration of the western region is the largest (0.7217), the East is the second (0.6657), and the central region is the smallest (0.6523).
4. The range of average betweenness of provinces and cities over the years is 0.0526–0.6009, and there are 14 provinces and cities play an important intermediary role in the regional high-end talent development efficiency network such as Tianjin, Anhui and so on. From the prospective of the three regions in China, the average betweenness of the central region is the largest (0.3780), the second is the East (0.3399), and the western region is the smallest (0.2698).
5. The path length evolutionary image of the regional high-end talent development efficiency network shows that the average path length of the network increases gradually over time. Therefore, with the introduction of the regional characteristics of high-end talent policies by provinces and cities, the correlation of high-end talent development efficiency shows a weakening trend in various provinces and cities, and the geographical characteristics of high-end talent are becoming more and more obvious.
